# Could Biomarkers of Bone, Cartilage or Synovium Turnover Be Used for Relapse Prediction in Rheumatoid Arthritis Patients?

**DOI:** 10.1155/2014/537324

**Published:** 2014-03-12

**Authors:** Delphine Dénarié, Elodie Constant, Thierry Thomas, Hubert Marotte

**Affiliations:** Department of Rheumatology, University Hospital Saint-Etienne, 42055 Saint-Etienne Cedex, France

## Abstract

*Objective*. The aim of this review is to clarify the usefulness of bone, cartilage, and synovial biomarker in the management of rheumatoid arthritis (RA) therapy in remission. *Synovial Biomarkers*. High MMP-3 levels are associated with joint progression in RA patients, but there is no data about their utility in clinical remission. IIINys and Glc-Gal-PYD seem to be more specific to synovium, but more studies are required. *Cartilage Biomarkers*. Unbalance between cartilage break-down biomarkers (urinary CTX II and COMP) and cartilage formation biomarker (PIIANP) was described. This unbalance is also associated with joint destruction and prognosis of destruction. No data are available on patients in remission. *Bone Biomarkers*. RA activity is correlated with an increase of bone resorption markers such as CTX I, PYD, and TRACP 5b and a decrease of bone formation markers such as OC and BALP. RA therapies seem to improve bone turnover in limiting bone resorption. There is no study about bone marker utility in remission. *Conclusion*. Biomarkers seem to correlate with RA activity and progression. They also could be used to manage RA therapies, but we need more data on RA remission to predict relapse.

## 1. Introduction

Rheumatoid arthritis (RA) is the most frequent chronic autoimmune inflammatory rheumatism, with a worldwide prevalence around 1% [1]. RA severity is related to joint destruction characterised by erosion and space narrowing that is responsible for joint functional disability [2–4]. Early diagnosis and treatment are crucial in order to prevent joint destruction and preserve joint function defining the “window of opportunity” concept [5, 6]. Since few years, the concept “Outside-Inside” suggested a beginning of RA disease also in the subchondral bone marrow [7]. In fact, a subchondral bone loss at the metacarpal phalangeal head starts since the early phase of RA disease [8]. Furthermore, joint inflammation due to synovitis is one of the most powerful predictors of new bone erosion [9]. So, the synovial membrane was the first actor mainly described by production of some mediators induced by inflammatory cytokines such as TNF or others. These mediators induced cartilage matrix degradation and subchondral bone loss [10, 11]. These data support a strong interaction between synovial membrane, cartilage, and subchondral bone. Inflammatory joint induced the release of specific protein fragments from its various compartments into the serum and the urine, which may be used as tissue specific biomarkers [12]. By this way, biomarkers of each component of the joint could be useful to manage RA patients.

TNF inhibitors and other biologics reduce synovitis, biomarkers of inflammation, and bone destruction. However, dissociation between clinical and radiological effect of TNF inhibitors has been reported. These TNF inhibitors are able to block joint destruction, even if RA disease is still active [13–15]. In 2014, in front of early RA patient, the goal of early RA therapy is to obtain remission according to the new criteria for remission ACR/EULAR [16]. However, though clinical remission was obtained, in some patients a structural progression can occur [17] probably due to persistence of joint inflammation [18, 19]. Exploration with specific biomarkers of each component of the joint could be helpful to investigate this paradigm [20].

In daily practice in 2014, only DAS28 combining clinical parameters with erythrocyte sedimentation rate (ESR) or C-reactive protein (CRP) is used. ESR and CRP are inflammatory biomarkers, but not specific to the joint. So, they are not strongly correlated with joint involvement. Despite its large usefulness in daily practice, DAS28 fails to strongly predict the joint progression or a real remission. At the time of “personalized medicine,” which aims to individually improve treatment management [21], biomarkers of the joint will be useful in RA especially at the early stage. The aim of this paper is to review some biomarkers of synovial, cartilage, and bone turnover in RA, clarify their utility in RA management, and analyze data in remission.

## 2. Synovial Biomarkers

Here, we focused our review on three major synovial biomarkers. Their interests to manage RA are summarized in Table 1.

Matrix metalloproteinase-3 (MMP-3 or stromelysin 1) is a proteinase secreted by synovial fibroblasts and chondrocytes. Its activity results in degradation of aggrecan core protein, cartilage link protein, fibronectin, and collagen types IV, VII, IX, and XI [22]. MMP-3 is present in RA synovial fluid and overexpressed in rheumatoid synovium [23, 24]. One MMP-3 polymorphism was described to be associated with higher joint damage in RA [25, 26]. Otherwise, serum MMP-3 level was suggested as a predictor for joint destruction in early RA [27, 28] or established RA [29, 30]. In fact, circulating MMP-3 level seems to be genetically determined [26]. Correlation between serum MMP-3 level and joint damage progression appeared to be independent of rheumatoid factor (RF) or ACPA status [31]. The next step was to assess MMP-3 variation induced by RA therapy and particularly during biological therapies. Anti-TNF therapy decreased MMP-3 expression in RA patients [32, 33]. Similar results were observed with tocilizumab (IL-6 blocker) [34] or abatacept (inhibitor of costimulation) [35]. Then, MMP-3 monitoring was investigated to improve therapeutic strategy. This was the purpose of the T-4 study [36]. The best outcome was observed in the group combining DAS28 and MMP-3 monitoring [36]. Finally, MMP-3 was also investigated in RA remission situation. Its level was similar in RA patients in remission or not induced by anti-TNF therapy [37]. However, normal MMP-3 level in RA patients treated with tocilizumab was predictive to absence of relapse after tocilizumab cessation [38]. To summarize, high MMP-3 level was associated with disease activity and joint progression in RA patients and should be used in association with usual inflammatory markers to follow therapy efficiency. However, this biomarker was never tested in patients in clinical remission to predict structural remission.

Another synovial biomarker considered in RA is the glycosylated form of pyridinoline (PYD) [39]. PYD is mainly a bone resorption biomarker but is also related to remodeling of cartilage and synovium [40]. The glycosylated analogue of PYD, glucosyl-galactosyl-PYD (Glc-Gal-PYD), can be assessed in urine and appeared to be specific to synovial tissue [39]. Urinary Glc-Gal-PYD level was higher in patients with early RA than controls and its high level is associated with higher risk for the progression of joint damage [28]. In established RA, urinary Glc-Gal-PYD was associated with changes of the erosion, joint space narrowing (JSN), and the total Sharp score [41]. After one year of anti-TNF therapy, the levels of urinary Glc-Gal-PYD was similar in RA patients with or without progressive joint damage over one year of anti-TNF therapy, but its reduction over one year was higher in patients with progressive joint damage [41]. These results suggested that, in some patients, other mechanisms were possibly involved than TNF related inflammation.

The last synovium biomarker recently developed is the nitrated type III collagen (IIINys), which was explored in both osteoarthritis (OA) and RA patients. In patients with joint disorder, the synovial membrane contains nitrated proteins [42]. IIINys was increased in serum from OA patients [43] and RA patients [44]. Its level was the highest in RA patients which suggests that it is related to synovial tissue inflammation [44]. However, no more data are currently available for this biomarker.

We attempted to describe synovial biomarkers and put out their interest in RA management. Despite many studies reviewed, no data are currently available to predict relapse in RA patients in remission. So, these biomarkers need to be tested in this situation.

## 3. Cartilage Biomarkers

Then, we focused on three main cartilage biomarkers with a summary of their characteristics in Table 2. Two are biomarkers of cartilage breakdown, whereas the third one is a biomarker of cartilage formation. Cartilage homeostasis consists in balance between degradation and formation. In RA, there is an imbalance in favour of destruction [45].

Cartilage is mainly composed of collagen type II (70%) and proteoglycans including aggrecan which is the most abundant one. MMPs and aggrecanases are mediators of cartilage degradation. Several cartilage degradation fragments can be measured. Collagen type II C-telopeptide (CTX-II) is a neoepitope generated from MMPs, derived from the carboxy-terminal part of type II collagen [46]. In early RA, urinary CTX-II level was higher than in controls, and patients with high CTX-II level have a higher risk for the progression of joint damage over 1 year, independent of the extent of joint destruction at baseline and of clinical indices of disease activity [28]. In established RA, urinary CTX-II level was associated with rapid radiologic progression [47] or changes of the JSN Sharp score over one year [41]. Then, CTX-II was assessed during anti-TNF therapy in RA patients. After one year of anti-TNF therapy, the levels of urinary CTX-II were similar in RA patients with or without progressive joint damage over one year of anti-TNF therapy. In patients with progressive joint damage, reduction of urinary CTX-II was higher than in others [41]. No data on RA remission are available at this time.

Cartilage oligomeric matrix protein (COMP) is a noncollagenous extracellular matrix protein mainly found in cartilage maintaining the integrity of the collagen network [48]. Serum COMP was reduced in RA patients in remission induced by anti-TNF therapy compared to other patients [37]. In early RA, early changes in serum COMP levels were related to radiological outcome over the first 5 years [49]. This biomarker was not yet analyzed during biologic therapy or in RA remission.

Serum propeptide of type IIA procollagen (PIIANP) arises from the maturation of type IIA procollagen. Thus, PIIANP is a biomarker of cartilage formation. Its level was decreased in patients with OA or RA. In RA patients treated with low-dose corticosteroids, serum PIIANP is significantly higher than in untreated patients [50]. No more data are currently available on biomarker of cartilage formation.

So unbalance between cartilage formation and breakdown is described in RA disease. No data are at this time available to describe their interest to predict relapse in RA patient in remission. More data are required in this situation to improve their utilities.

## 4. Bone Biomarkers

Bone homeostasis is highly regulated by balance between new bone formation and removing old bone. Activated osteoclasts degrade bone matrix while osteoblasts form new matrix [51]. Type I collagen constitutes 90% of bone matrix. Bone formation markers included the serum bone formation markers total osteocalcin (OC), the alkaline phosphatase bone isoenzyme (BALP), and the C- and N-propeptide of type I collagen (PICP and PINP). Bone degradation is driven by osteoclasts and results in stimulation by RANKL induced by IL-1*β*, IL-6, or TNF. Osteoclasts secrete cathepsin K, which degrades the collagen type I and releases C-terminal crosslinked telopeptide of type I collagen (CTX-I), or N-terminal crosslinked telopeptide of type I collagen (NTX) neoepitope. The crosslinked carboxyterminal telopeptide of type I collagen (ICTP) is another fragment of C-telopeptide end, which is not released with cathepsin K action but probably MMPs [52, 53]. Other type I collagen crosslinks are pyridinoline (PYD) and deoxypyridinoline (DPD) [54].

In established RA, uncoupling with low level of bone formation markers and high bone resorption markers was described in 1999 [55]. OC, a bone formation marker, was reduced in RA without destruction compared to controls. On the contrary, CTX-I, a catabolic bone marker, is higher in RA patients with destruction compared to other RA patients [55]. This uncoupling was recently confirmed by using an innovative way to assess bone damage in RA by high-resolution peripheral quantitative computed tomography (HR-pQCT) [56]. TRAP 5b level, a catabolic bone marker, was associated with bone erosions, whereas bone alkaline phosphatase (BAP) was associated with osteophytes [57]. Furthermore, in longitudinal studies, catabolic bone markers (CTX-I or PYD) are also good predictors for radiologic progression in RA [47, 58, 59].

Like cartilage and synovium turnover markers, bone biomarkers were assessed during various biological therapies. During anti-TNF therapy, ratio between bone formation markers and bone resorption markers increased during one year of treatment, suggesting improvement of the bone remodeling balance, mainly due to a decrease in bone resorption [60]. A differential effect was observed at one year of anti-TNF therapy between ICTP and CTX-I. ICTP, which is related to MMPs activity, remained decreased at one year, whereas CTX-I level, which is related to cathepsin K, returned to its baseline level at one year [60]. This suggests a strong effect of anti-TNF on local subchondral bone related to joint inflammation. Since TNF blockers already showed a reduction of the bone biomarker unbalance, TNF blockers also demonstrated a positive effect on bone mineral density in RA patients with or without a clinical response as observed at the joint level [61]. Serum RANKL was decreased during anti-TNF therapy [62]. All these data support that anti-TNF therapy is not only able to prevent joint destruction, but it is also able to prevent bone loss in RA patients. Similarly, with tocilizumab, bone formation biomarker PINP increased whereas bone resorption markers, ICTP and CTX-I, decreased [63]. So TNF or IL-6 inhibitors increased bone formation/bone resorption ration. This suggests a nonspecific effect of a pathway but an effect on suppression of joint inflammation. Denosumab is also a biotherapy targeting RANKL [64], but not a proinflammatory cytokine. Denosumab reduced both serum PINP and CTX-I levels over one year [65], whereas urinary CTX-II decreased only at 3 months. Since denosumab targets RANKL, but not a proinflammatory cytokine, RA disease was not improved, but it reduced erosion progression. So according to the target, drugs have different effects. Blocking inflammation reduces bone loss, but blocking pathway induced in bone loss reduced it without effect on RA activity.

Among all these biomarkers, only CTX-I has demonstrated its ability to be associated with joint destruction, sensitivity to treatment, and prediction of joint progression. However, no data are available for relapse prediction in RA remission.

## 5. Discussion

We showed that synovium, cartilage, and bone turnover biomarkers are correlated with RA activity. To summarize, resorption markers increase with RA activity in the three components of the joint. Furthermore, these biomarkers could be useful to identify RA patients with high risk of rapid disease progression. This suggests that these selected RA patients require a rapid active therapy. Since these biomarkers reflected different compartments involved in RA, they will be useful to define structural remission in RA. Some of these reviewed biomarkers compose the multibiomarker disease activity (MBDA) test developed to quantify RA disease activity [66]. Recent data suggested that low MBDA was associated with clinical remission criteria [67, 68]. However, no study currently explored MBDA to predict relapse in RA remission. Treat-to-target strategy emerged since few years to manage early RA patients. This strategy aims to achieve clinical remission and appears to be a realistic today [69]. Only one study combining clinical and biomarkers demonstrated its utility in the treat-to- target strategy [36]. This study is the typical example of the “personalized medicine” [70]. The only biomarker with enough promising results is MMP-3. However, we need more studies to generate more data to define the place of these biomarkers in RA remission. At this time, we failed to have the “perfect” biomarker which could be used in RA management such as HbA1c in diabetes [71].

## Figures and Tables

**Table 1:** Synovium biomarkers and their interests in RA management.

Synovial biomarker	Expressed in RA	Treatment response	Joint destruction	Effects on monitoring in clinical response and progression
MMP-3	[23, 24]	[32–35]	[25, 27, 29–31]	[36]
Glc-Gal-PYD	[28, 39, 40]	No data available	[28, 39, 41]	No data available
IIINys	[44]	No data available	No data available	No data available

MMP-3: matrix metalloproteinase-3; Glc-Gal-PYD: glucosyl-galactosyl pyridinoline; IIINys: nitrated type III collagen.

**Table 2:** More studied cartilage biomarkers and their interests in RA management.

Cartilage biomarker	Expressed in RA	Treatment response	Joint destruction
CTX-II	[28, 47]	[41]	[28, 41, 47]
COMP	[49]	[37]	[49]
PIIANP	[50]	No data available	No data available

CTX-II: collagen type II C-telopeptide; COMP: cartilage oligomeric matrix protein; PIIANP: propeptide of type IIA procollagen.
